# Identifying Rare FHB-Resistant Segregants in Intransigent Backcross and F_2_ Winter Wheat Populations

**DOI:** 10.3389/fmicb.2016.00277

**Published:** 2016-03-07

**Authors:** Anthony J. Clark, Daniela Sarti-Dvorjak, Gina Brown-Guedira, Yanhong Dong, Byung-Kee Baik, David A. Van Sanford

**Affiliations:** ^1^Department of Plant and Soil Sciences, University of KentuckyLexington, KY, USA; ^2^Syngenta SeedsDes Moines, IA, USA; ^3^Plant Science Research Unit, United States Department of Agriculture - Agricultural Research ServiceRaleigh, NC, USA; ^4^Department of Plant Pathology, University of MinnesotaSaint Paul, MN, USA; ^5^Soft Wheat Quality Laboratory, United States Department of Agriculture - Agricultural Research ServiceWooster, OH, USA

**Keywords:** resistance breeding, Fusarium head blight, deoxynivalenol, soft red winter wheat, marker-assisted selection

## Abstract

Fusarium head blight (FHB), caused mainly by *Fusarium graminearum* Schwabe [telomorph: *Gibberella zeae* Schwein.(Petch)] in the US, is one of the most destructive diseases of wheat (*Triticum aestivum* L. and *T. durum* L.). Infected grain is usually contaminated with deoxynivalenol (DON), a serious mycotoxin. The challenge in FHB resistance breeding is combining resistance with superior agronomic and quality characteristics. Exotic QTL are widely used to improve FHB resistance. Success depends on the genetic background into which the QTL are introgressed, whether through backcrossing or forward crossing; QTL expression is impossible to predict. In this study four high-yielding soft red winter wheat breeding lines with little or no scab resistance were each crossed to a donor parent (VA01W-476) with resistance alleles at two QTL: *Fhb1* (chromosome 3BS) and *QFhs.nau-2DL* (chromosome 2DL) to generate backcross and F_2_ progeny. F_2_ individuals were genotyped and assigned to 4 groups according to presence/ absence of resistance alleles at one or both QTL. The effectiveness of these QTL in reducing FHB rating, incidence, index, severity, *Fusarium*-damaged kernels (FDK) and DON, in F_2_-derived lines was assessed over 2 years. *Fhb1* showed an average reduction in DON of 17.5%, and conferred significant resistance in 3 of 4 populations. *QFhs.nau-2DL* reduced DON 6.7% on average and conferred significant resistance in 2 of 4 populations. The combination of *Fhb1* and *QFhs.nau-2DL* resistance reduced DON 25.5% across all populations. Double resistant lines had significantly reduced DON compared to double susceptible lines in 3 populations. Backcross derived progeny were planted in replicated yield trials (2011 and 2012) and in a scab nursery in 2012. Several top yielding lines performed well in the scab nursery, with acceptable DON concentrations, even though the average effect of either QTL in this population was not significant. Population selection is often viewed as an “all or nothing” process: if the average resistance level is insufficient, the population is discarded. These results indicate that it may be possible to find rare segregants which combine scab resistance, superior agronomic performance and acceptable quality even in populations in which the average effect of the QTL is muted or negligible.

## Introduction

Fusarium head blight (FHB), caused by several *Fusarium* species, is a destructive disease of wheat (*Triticum aestivum* L. and *T. durum* L.) and barley (*Hordeum vulgare* L.) worldwide (Bai and Shaner, 1994; Mesterhazy, 1995). In North America, *Fusarium graminearum* was primarily responsible for scab epidemics since 1993 in the spring, soft red winter and hard red winter wheat regions of the US. It is estimated, based on published literature (Windels, 2000; Johnson et al., 2003; Nganje et al., 2004) and anecdotal reports from millers, pathologists and breeders, that direct losses to scab in the US from 1993 to 2014 total $ 4.8 B. As Nganje et al. (2004) note, the number would be increased by the inclusion of secondary (indirect) losses. Many wheat breeding programs focus on, along with high yield, the development of FHB resistance in commercial cultivars (McMullen et al., 2012). The incorporation of genetic resistance reduces the need for fungicide applications and, consequently, reduces production costs and environmental pollution while increasing food safety.

A major concern associated with FHB in wheat and barley is the production of mycotoxins, especially deoxynivalenol (DON) and its derivatives. High levels of DON in grains have negative effects on animal production, causing vomiting in non-ruminant animals leading to serious feeding problems and economic losses (McMullen et al., 1997; Pestka, 2007). There is support for the premise of a close linear relationship between FHB resistance and DON concentration in the infected grain (Mesterházy et al., 1999). Regulation of DON accumulation is challenging and depends on the host and fungal genotypes as well as environmental conditions (Mesterházy et al., 1999). Deoxynivalenol concentration is also the most important FHB trait because of the discount imposed on contaminated grain at the elevator. FDK is another indicator of kernel damage and relates to test weight reduction and partly accounts for yield reduction. FHB index is the best overall indicator of field symptoms and is a product of severity and incidence. Ratings theoretically should be very similar to index when given by very experienced workers and are attractive because they can be recorded quickly. Near infrared reflectance (NIR) measurements can be an efficient way to measure grain symptoms.

One of the major discussions within the US soft red winter (SRW) wheat community concerns the use of exotic (i.e., from outside the pool of elite US lines) resistance QTL (e.g., *Fhb1*) vs. so-called “native” resistance found in adapted SRW wheat cultivars and breeding lines. The existence or extent of linkage drag associated with exotic FHB resistance QTL on agronomic traits has not been widely documented but each new variety, with improved yield over its predecessors, represents the pinnacle of development and testing of thousands of new segregants. If linkage drag were present, exotic QTL-containing lines would likely be out yielded. The genetics of native resistance are largely unknown, and likely involve numerous genes of small effect. While the exotic QTL are easy to track with DNA markers, the level of FHB resistance conferred by a single QTL is often insufficient to reduce losses in grain yield and quality. Further, QTL by environment interactions and the effects of different genetic backgrounds on gene expression complicate the story (Van Sanford et al., 2001; Balut et al., 2013).

Previous research in our lab has shown that two FHB resistance QTL, *Fhb1* and *QFhs.nau-2DL* vary in their expression levels according to genetic background (Verges et al., 2006; Agostinelli et al., 2012; Balut et al., 2013). This finding is in accord with other research on *Fhb1* (Pumphrey et al., 2007; Buerstmayr et al., 2009) and *QFhs.nau-2DL* (Jiang et al., 2007a,b) and the 2DL QTL studied by Kang et al. (2011). In a single population study, Agostinelli et al. (2012) reported that *QFhs.nau-2DL* was more effective than *Fhb1*, reducing FDK and DON by 40 and 55%, respectively, in comparison to *Fhb1*-associated reductions of 32% for FDK and 25% for DON. Balut et al. (2013) observed that *Fhb1* reduced FDK by 32%, and DON concentration by 20% across five populations and *QFhs.nau-2DL* reduced FDK and DON by as much as 29 and 24%, respectively in some backgrounds. The lines chosen for the latter study were all homozygous for *Fhb1* but while the populations segregated for *QFhs.nau-2DL* many of the lines tested derived from heterozygotes. The current study is the first to look at the effect of *QFhs.nau-2DL* in several populations using lines homozygous at that locus.

The constraints associated with the exotic QTL, e.g., dependence on genetic background, plus the need to combine exotic QTL-based resistance with native resistance, are daunting. These issues do not encourage a resource-intensive backcrossing effort, lest the recurrent parent be in the wrong background or in a background devoid of native resistance. This study was undertaken to evaluate an approach to population development that might yield useful segregants independent of genetic background.

The objectives of the study were to: (1) determine whether useful segregants for FHB resistance and agronomic fitness could be recovered from populations in which overall resistance was not high enough to recommend backcrossing, and (2) whether F_2_ or BC_1_F_1_ populations were superior sources of such segregants.

## Materials and methods

### Initial population development

The study began as a marker-assisted backcrossing project in which FHB resistance alleles from two QTL, *Fhb1* and *QFhs.nau-2DL*, from VA01W-476 were introgressed into four elite SRW breeding lines, KY97C-0321-05-2, KY97C-0519-04-05, KY97C-0540-01-03, and KY97C-0508-01-01A. These recurrent parents were high yielding, FHB-susceptible wheat lines. The FHB-resistance donor, VA01W-476, was a double haploid line derived from “Roane” and “W14” (Perugini, 2007; Agostinelli et al., 2012; Balut et al., 2013). VA01W-476 in addition to providing resistant *Fhb1* and *QFhs.nau-2DL* alleles, also likely has additional native resistance genes from its parents (Jiang et al., 2007a; Agostinelli et al., 2012). All crosses used in this study were made by A.J. Clark.

The intent was to use genotyped F_2_—derived lines to validate the resistance QTL while at the same time examine and compare the agronomic and quality characteristics of a separate set of BC_1_F_1_ and F_2_ derived lines from the same four crosses that had undergone agronomic selection during their development.

### F_2_-derived populations used in QTL validation study in scab nursery

Single crosses were made in the greenhouse in spring 2008. KY97C-0321-05-2/VA01W-476 was developed as population 2, KY97C-0519-04-05/VA01W-476 as population 3, KY97C-0540-01-03/VA01W-476 as population 4 and KY97C-0508-01-01A/VA01W-476 as population 6. Three additional backcrossed populations, 1, 5, and 7 were made but F_2_ lines were not derived from them. Populations 2, 3, 4, and 6 were advanced to the F_2_ generation in a greenhouse at Spindletop Research Farm, near Lexington, KY, in the fall of 2008 and F_2:3_ and F_2:4_ lines tested in the scab nursery.

### Development of populations used in yield trials and for quality testing

#### Agronomic BC_1_F_1−_ derived population development

After crosses between recurrent and donor parents were made, plants positive for the presence of resistance alleles at both loci were selected and crossed back to the recurrent parent. BC_1_F_1_ seedlings were grown in the greenhouse and BC_1_F_1_ heads harvested and planted in BC_1_F_1:2_ head-rows in 2009 at Spindletop Research Farm near Lexington, KY. For populations 2, 3, 4 and 6, 65, 23, 42 and 32 selections were made and thoserows harvested and threshed and BC_1_F_1:3_ and BC_1_F_1:4_ tested in 2011 and 2012 respectively. These BC_1_F_1:4_ populations were also characterized in the scab nursery in 2012.

#### Agronomic F_2_-derived population development

Tillers of the F_1_ plants produced in 2008 were allowed to self-fertilize, to produce F_2_ seed. Heads were threshed in bulk and planted in F_2_ plots in Lexington, KY, 2009. From each population approximately 60–100 heads were selected and planted the following year in 1.2 m long F_2:3_headrows, spaced 30 cm apart, in Princeton, KY. Headrow selections were made for agronomic potential and threshed independently. For populations 2, 3, 4, and 6, 21, 19, 44 and 36 F_2:4_ lines were selected respectively.

### Genotyping

DNA was isolated according to Pallota et al. (2003). Markers used were UMN10 (Liu et al., 2008) and gwm533 (Röder et al., 1998) for *Fhb1*; and cfd233 (Grain genes 2.0 at http://wheat.pw.usda.gov/GG3/ verified Nov 19th 2015) and gwm539 (Röder et al., 1998) for *QFhs.nau-2DL*. The genotyping process was divided between two laboratories: the University of Kentucky Wheat Breeding Laboratory and the USDA/ARS Regional Small Grains Genotyping Lab (RSGGL) (http://www.ars.usda.gov/Main/docs.htm?docid=19522) at Raleigh, NC. At the University of Kentucky, PCR products were separated using an ABI 3730 DNA Analyzer (Applied Biosystems) and sized using GeneMapper v4.0. Following backcrossing seedlings heterozygous for both *Fhb1* and *QFhs.nau-2DL* resistant alleles were selected for growth in the greenhouse. For QTL validation, F_2_ seedlings homozygous for markers at both QTL were selected in all combinations, RR, RS, SR, SS, at *Fhb1* and *QFhs.nau-2DL* respectively.

### Scab nursery

For the 2011 season, 78, 79, 131, and 91 lines from Populations 2, 3, 4, and 6, respectively, along with parents were planted in replicated F_2:3_ headrows in a misted, inoculated scab nursery at the UK Spindletop Research Farm (38°7′37.81″N, 84°29′44.85″W; Maury silt loam [fine, mixed, semiactive, mesic Typic Paleudalfs]) near Lexington, KY on 11 October, 2010. Each row was evaluated for FHB traits, harvested by row, and screened for grain disease levels. The following year, F_2:4_ headrows were planted in the scab nursery on 17 October, 2011. The experiment planted each year was a randomized complete block design (RCBD) with 2 replications. In addition in 2012 the BC_1_F_1:4_ lines used for agronomic testing were also seeded in 2 rep RCBD experiments in the Lexington scab nursery. Unfortunately space constraints prevented inclusion of the F_2_-derived lines used for agronomic testing. Rows were 1 m long, spaced 30 cm apart. Rows were misted with an overhead mist irrigation system on an automatic timer, from May to June, for periods of 5 min, every quarter hour from 8:00 pm to 8:45 pm, 11:00 pm to 11:45 pm, 2:00 am to 2:45 am, 5:00 am to 5:30 am, and for one time at 8:30 am so the integrity of the system could be monitored.

The scab nursery was inoculated with *Fusarium graminearum*—infected corn (*Zea mays* L.) (Verges et al., 2006). Inoculum source and preparation were exactly as described in Balut et al. (2013). The scabby corn was distributed between rows at a rate of 11.86 g/m^−2^, approximately 3 week prior to heading, on 14 April and 31 March of 2011 and 2012, respectively. Liquid nitrogen fertilizer (28% UAN) was applied in the spring at a rate of 105 kg N/ha in split applications. Harmony Extra herbicide was applied on 20 April 2011 and 20 March 2012.

### Phenotyping

Heading dates were recorded for each headrow in the scab nursery, when 50% of the spikes in the row had emerged from the flag leaf sheath. Plant height was measured at the soft dough stage. Effectiveness of QTL in reducing FHB was assessed through several resistance traits. These traits were measured approximately 21 days after anthesis and consisted of: rating, severity, incidence, and FHB index. Ratings were visual estimates on a 0–9 scale, where 0 = 0–10% and 9 = 91–100% of diseased spikelets within a row. Incidence was the count of spikes showing any disease among 20 randomly selected spikes in a headrow, expressed as a percentage. Severity, the number of visually infected spikelets divided by the total number, expressed as a percentage was also counted, in 10 randomly selected blighted heads per row. FHB index is the product of severity and incidence divided by 100.

Each headrow was hand harvested with a sickle and threshed in a small thresher with low air flow to avoid loss of tombstones (infected kernels, blighted, and lighter than healthy grains). Fusarium damaged kernels (FDK) percentages were estimated from carefully cleaned samples run through an air separation machine (Agostinelli, 2009; Agostinelli et al., 2012). FDK was expressed as the weight of scabby kernels divided by total weight. DON concentrations were estimated in these same samples by the University of Minnesota DON Testing Laboratory using gas chromatography with mass spectrometry (GC-MS) (Mirocha et al., 1998; Fuentes et al., 2005).

### Near-infrared reflectance spectroscopy

Near-Infrared Reflectance Spectroscopy (NIR) predictions of FDK and DON were also generated (Delwiche and Hareland, 2004) using the Perten Instruments DA7200. We ran samples of cleaned grain (15–20 g) through the instrument to predict FDK and DON (FDKNIR, DONNIR, respectively) and compared predictions with actual values.

### Data analysis of QTL validation study

Analysis of variance (ANOVA) was performed using the General Linear Model procedure (PROC GLM; SAS, 2011). The model used was:

$$\begin{array}{l} {\text{Y}}_{\text{ij}}=\mu +{\text{ENV}}_{\text{i}}+\text{R}{\left(\text{ENV}\right)}_{\text{ij}}+\text{QTL}+{\text{G}}_{\text{k}}(\text{QTL})\\ \;+{\text{ENV}}_{\text{i}}{}^{*}\text{QTL}+{\text{E}}_{\text{ij}} \end{array}$$

Where:

Y_ij_ = observation in the kth genotype in the jth rep in the ith environment,

μ = overall mean,

G_k_(QTL) = effect of the kth genotype within QTL,

QTL = effect of the QTL,

R(ENV)ij = effect of jth rep within ith environment,

ENVi ^*^ QTL = effect of the interaction of the ith environment with the QTL,

E_ij_ = residual error.

Fisher's Least Significant Difference (LSD) was used to corroborate significant differences among QTL combination classes.

Broad sense heritability of FHB and agronomic traits estimates were based on entry means using the following model:

$$\text{Yij}=\;\mu +\text{ENVi}+\text{R}(\text{ENV})\text{ij}+\text{Gk}+{\text{Gk}}^{*}\text{ENVi}+\text{Eij}$$

Where:

Y_ij_ = the observation in the k^th^ genotype in the j^th^ rep in the i^th^ environment,

μ = the overall mean,

G_j_ = the effect of the k^th^ genotype,

R(ENV)_ij_ = the effect of j^th^ rep within i^th^ environment,

G_k_^*^ ENV_i_ = the effect of the interaction of the k^th^ genotype with the i^th^ environment,

E_ij_ = the residual error.

Data were analyzed using PROC GLM (SAS, 2011). Genotypic and phenotypic variances were estimated from the expected mean squares (EMS) and heritability estimates were computed as:

$${\text{h}}^{2}={\text{V}}_{\text{g}}{\text{/V}}_{\text{p}}$$

Where:

h^2^ = broad sense heritability,

V_g_ = genotypic variance,

V_p_ = phenotypic variance.

Confidence intervals (90%) for the heritability estimates were calculated after Knapp et al. (1985).

PROC CORR (SAS, 2011) was used to analyze the relationship among traits on an entry mean basis. Multiple comparisons of least squares means presented in **Tables 3–5** were handled by performing a *t*-test on every pair of means.

### Yield measurement

BC_1_F_1:3_ plots were planted on 13th October 2010 in a randomized complete block design, with two replications, at Spindletop Farm, near Lexington, KY. In 2011, BC_1_F_1:4_ plots along with F_2:4_ plots were planted in a randomized complete block design, with two replications at two locations, Lexington, KY (1 Nov. 2011) and Princeton, KY (10 Oct. 2011). Each population's parents were planted along with four commercial varieties as yield checks. The experimental material was grown in conventional yield plots 6 rows wide and 3 m long, with a row spacing of 17.8 cm. All plots received 105 kg ha^−1^ of actual N applied in the spring; recommended wheat production practices for Kentucky were followed (Lee et al., 2009). Plant height, yield and test weight were measured in each plot.

### Milling and baking quality

In 2012, a 100-g sample from each replication was analyzed for milling and baking quality at the USDA-ARS Soft Wheat Quality Laboratory, Wooster, OH. Grain was tempered to 15% moisture before milling and milled using a Brabender Quadrumat Senior laboratory mill (South Hackensack, NJ). Flour yield was determined as the percent total flour weight (break flour + middlings) over tempered grain weight. Softness equivalent was the percentage weight break flour over the total flour (break flour + middlings). Water SRC, sucrose SRC, sodium carbonate SRC, and lactic acid SRC were determined using approved AACC International method 56-11.02 (American Association of Cereal Chemists, 2010) and were used to calculate the gluten performance index (GPI), defined as GPI = lactic acid SRC/(sodium carbonate SRC + sucrose SRC), as described by Kweon et al. (2011).

## Results

### Weather conditions and disease levels

Weather conditions during 2011 were favorable for scab development, while unusually warm temperatures in 2012 from March through May accelerated wheat growth and reproductive development and resulted in heading dates 3–4 weeks earlier than normal. A severe April 2012 freeze followed by below normal rainfall led to drought-like conditions which minimized disease pressure; natural scab levels were much lower in Kentucky than in 2011 (Bruening et al., 2012). A uniform disease epidemic was achieved in the irrigated nursery but the level was also lower in 2012 (Table 1). Weather and scab intensity differences in any two growing seasons are not uncommon in Kentucky; 2011 and 2012 allowed us to observe QTL effects in very different environments.

**Table 1:** **Range of FHB traits in four F_2_ derived wheat populations and their parents, Lexington, KY, 2011 and 2012**.

**Entry**	**N**	**DON**	**FDK**	**Sev**	**Inc**	**Ind**	**Rating**	**Heading**
		**ppm**	**__________ % __________**				**0–9**	**Julian days**
**2011**								
Population 2	78	5–26	5–49	29–71	19–88	7–62	1.5–7.5	130–144
VA01W476	1	7	11	36	39	14	2.0	129
KY97C-032	1	28	32	63	73	46	3.0	138
LSD (0.05)	80	8.9	21.8	18.8	32.8	23.8	2.4	5.7
Population 3	79	5–27	7–58	34–70	30–93	11–57	1.0–7.0	131–141
VA01W476	1	5	9	38	43	16	2.5	131
KY97C-519	1	41	40	57	95	54	7.0	137
LSD (0.05)	81	7.6	17.2	21.2	32.8	23.0	2.2	6.3
Population 4	131	6–34	5–71	25–71	15–83	5–53	1.5–7.0	130–143
VA01W476	1	10	11	30	60	18	2.0	130
LSD (0.05)	132	11.6	30.2	21.3	33.4	22.4	2.4	5.7
Population 6	91	6–27	8–52	18–59	23–76	7–42	1.0–7.0	128–139
VA01W476	1	6	10	30	33	10	2.0	130
KY97C-050	1	20	37	29	33	11	5.0	131
LSD (0.05)	93	5.7	21.1	18.0	26.4	15.1	2.4	4.2
**2012**								
Population 2	78	2–24	3–19	8–44	15–90	2–36	0.5–9.0	107–120
VA01W476	1	2	5	14	30	4	5	108
KY97C-032	1	28	16	29	68	20	0.5	118
LSD (0.05)	80	9.4	5.8	12.9	30.0	11.7	2.7	3.6
Population 3	79	1-10	3-16	7-36	10-63	1-21	0.0-6.0	106-114
VA01W476	1	1	7	17	23	5	2.0	108
KY97C-519	1	12	19	29	75	22	7.5	115
LSD (0.05)	81	3.2	4.8	9.7	27.8	6.4	2.2	1.8
Population 4	131	1–18	3–14	6–36	10–70	1–20	0.5–7.5	106–117
VA01W476	1	2	13	12	55	8	1.5	107
KY97C-054	1	9	6	30	65	20	7.0	118
LSD (0.05)	133	4.7	4.0	25.3	27.6	7.1	2.1	2.0
Population 6	91	1–11	5–19	7–54	5–68	1–32	0.0–5.0	103–119
VA01W476	1	3	11	18	33	9	1.5	108
KY97C-050	1	5	15	8	15	1	1.5	109
LSD (0.05)	93	4.5	7.1	17.2	29.0	12.4	2.3	2.3

Recurrent parent in population 4, KY97C-054, not planted 2011.

DON, dexynivalenol; FDK, Fusarium-damaged kernels; Sev, severity; Inc, incidence; Ind, index; Rating, Fusarium head blight rating.

In both years of the study, susceptible parents showed higher disease levels than the resistant parent in all populations. F_2_ derived progeny with DON levels lower than the resistant parent were observed in three populations in 2011 and four populations in 2012 (Table 1).

### Heritability estimates

Broad sense heritabilities and their corresponding 90% confidence intervals were estimated on an entry mean basis for each population separately and also for all populations combined to provide an overall heritability for each disease trait (Table 2). DON h^2^ estimates were relatively high and consistent and ranged from 0.54 to 0.75. FDK h^2^ ranged from 0.16 tp 0.48. Incidence h^2^ was moderate ranging from 0.34 to 0.57 while heritability of severity was ≥0.30. FHB index h^2^ estimates were moderate, with an average of 0.40. Heritabilities of FHB ratings varied widely across populations from 0.19 to 0.65 (Table 2).

**Table 2:** **Heritabilities and their 90% confidence interval (in parentheses) of four F_2_ derived wheat populations based on 2 year entry means, Lexington, KY 2011-2012**.

	**DON**	**FDK**	**Incidence**	**Severity**	**Index**	**Rating**
Overall	0.65 (0.60–0.69)	0.32 (0.23–0.41)	0.51 (0.44–0.57)	0.24 (0.14–0.34)	0.41 (0.33–0.48)	0.37 (0.28–0.44)
Pop2	0.66 (0.54–0.74)	0.41 (0.21–0.56)	0.49 (0.32–0.62)	0.14 (-0.14–0.36)	0.42 (0.23–0.57)	0.19 (-0.08–0.39)
Pop3	0.54 (0.38–0.95)	0.48 (0.31–0.61)	0.42 (0.22–0.56)	0.30 (0.07–0.47)	0.35 (0.14–0.51)	0.48 (0.31–0.61)
Pop4	0.75 (0.69–0.80)	0.16 (0.05–0.33)	0.57 (0.47–0.66)	0.26 (0.07–0.41)	0.43 (0.29–0.54)	0.36 (0.20–0.48)
Pop6	0.61 (0.49–0.70)	0.38 (0.19–0.53)	0.34 (0.14–0.66)	0.26 (0.41–0.66)	0.43 (0.25–0.56)	0.65 (0.54–0.73)

### QTL effects on DON, FDK, and FHB disease traits

#### Fhb1

Under heavy scab pressure in 2011 and reduced pressure in 2012, the effectiveness of *Fhb1* in reducing DON varied among populations. In populations 3, 4, and 6 significant (*P* ≤ 0.05) DON reductions were observed in both 2011 and 2012 ranging from 11 to 32% (Table 3). Significant (*P* ≤ 0.05) ENV^*^QTL interactions were seen for DON accumulation for populations 4 and 6. In both populations the percentage reduction shown by *Fhb1* resistant lines was higher in 2012 when overall DON levels were lower. In population 3, resistant lines were lower in 2012 than in 2011 but there was no significant ENV^*^QTL effect. In population 2, resistance alleles at this QTL had no effect on DON level (Table 3). Natural field infections typically result in disease pressure similar to that in the inoculated scab nursery in 2012; therefore one can expect *Fhb1* to be effective in reducing typical DON levels in farmers' fields. FDK was significantly reduced in both years in populations 4 and 6 (Table 3). Compared to the widespread and consistent-between-years differences between resistant and susceptible *Fhb1* lines in grain symptoms, differences in detailed head symptoms were less frequent or consistent. For example, a significant (*P* < 0.05) reduction in incidence and index was seen in population 4 in 2012 but not 2011 (Table 3). Population 2 rating, based on the overall row saw a significant increase in *Fhb1* resistant lines in 2011 only. The lack of consistency between the patterns of incidence/severity/index and FDK/DON show that the former should not substitute for grain measurements. In 2012 *Fhb1* resistant lines were significantly (*P* < 0.05) earlier however this was not seen in 2011.

**Table 3:** **Means for FHB traits evaluated according to the presence of resistance (R) or susceptible (S) alleles at *Fhb1* for four F_2_ derived wheat populations, Lexington, KY, 2011 and 2012**.

**Population**	**Allele**	**N**	**DON**	FDK^*^	**Inc**	**Sev**	**Ind**	**Rating**	**Heading**
			**ppm**	**__________%___________**				**0–9**	**Julian days**
**2011**									
2	R	40	13.7	22.4	57.3	51.4a	29.9a	4.2a	133.8
	S	38	14.3	22.7	52.7	47.3b	25.9b	3.7b	134.7
3	R	39	12.0b	23.1	59.4	48.3	29.2	4.3	134.1
	S	40	13.5a	23.5	56.8	51.5	29.7	4.2	134.0
4	R	67	12.5b	20.6b	49.0	46.5	23.4	3.7b	133.9
	S	64	16.1a	27.6a	52.4	47.9	25.6	4.2a	134.4
6	R	45	11.6b	21.9b	42.6	35.9	16.1	3.4b	131.7
	S	46	15.0a	25.7a	45.3	38.0	17.7	4.4a	131.2
**2012**									
2	R	40	8.2	9.5	51.6	18.4	10.3	3.4	110.2b
	S	38	8.4	9.8	53.8	20.3	12.0	3.7	113.1a
3	R	39	3.2b	8.5	32.5	13.0	4.8	2.0	109.9
	S	40	3.7a	8.1	35.9	13.9	5.4	2.2	109.8
4	R	67	3.6b	6.2b	31.5b	12.5	4.4b	1.8b	109.2
	S	64	5.3a	7.7a	36.9a	12.8	5.3a	2.5a	109.5
6	R	45	3.9b	9.0b	31.2	15.7	5.9	1.4b	107.2
	S	46	5.4a	10.4a	30.2	16.4	5.7	2.0a	107.1

* FDK, Fusarium damaged kernels; Sev, severity; Inc, incidence; Ind, FHB index.

#### QFhs.nau-2DL

*QFhs.nau-2DL* was similar to *Fhb1* in that DON levels were significantly reduced in populations 3 (21%), 4 (19%), and 6 (18%) in 2012; though we only observed a significant reduction in population 6 in 2011 (Table 4). Unlike *Fhb1* resistance, the largest DON reduction was observed in Population 3 in 2012, where DON levels were 21% lower in *QFhs.nau-2DL* resistant lines (Table 4). As we observed with *Fhb1*, the R allele at *QFhs.nau-2DL* did not reduce DON in population 2 (Table 4). In the other populations, *QFhs.nau-2DL* effects ranged from 14 to 17% varying across years (Table 4). As we noted with *Fhb1*, effects of *QFhs.nau-2DL* on incidence, severity and index were scarce and inconsistent between years. In contrast to *Fhb1* there were no consistent effects on rating with only population 2 showing a significant (*P* < 0.05) benefit in 2011 and the opposite in 2012 (Table 4). Average heading dates were significantly (*P* < 0.05) increased in resistant lines of populations 3 (0.9 days), 4 (0.4 days), and 6 (0.6 days) in 2012 when heading was unusually early (Table 4). None of these differences was seen in the more typical 2011.

**Table 4:** **Means for FHB traits evaluated according to the presence of resistance (R) or susceptible (S) alleles at *QFhs.nau-2DL* for four F_2_ derived wheat populations, Lexington, KY, 2011 and 2012**.

**Population**	**Allele**	**N**	**DON**	**FDK**	**Inc**	**Sev**	**Ind**	**Rating**	**Heading**
			**ppm**	**___________%___________**				**0–9**	**Julian days**
**2011**									
2	R	35	13.6	20.6	55.4	46.9b	26.8	3.6b	134.7
	S	43	14.3	24.1	54.8	51.4a	28.8	4.3a	133.8
3	R	34	12.7	20.7b	56.5	48.2	27.4	4.1	134.1
	S	45	12.9	25.2a	59.2	51.1	30.9	4.3	134.0
4	R	44	13.5	21.6	51.0	46.9	24.5	3.9	134.1
	S	87	14.6	25.2	50.5	47.4	24.4	4.0	134.2
6	R	39	12.7b	21.3b	43.6	34.6b	15.8	3.8	131.2
	S	52	13.7a	25.7a	44.2	38.7a	17.8	4.0	131.7
**2012**									
2	R	35	8.5	9.8	53.0	19.9	12.0	3.8a	111.2
	S	43	8.0	9.6	52.3	18.8	10.5	3.3b	112.2
3	R	34	3.0b	7.5b	29.6b	13.1	4.6	2.0	110.4a
	S	45	3.8a	8.9a	37.7a	13.7	5.5	2.2	109.5b
4	R	44	3.8b	6.2b	32.5	13.0	5.0	2.2	109.6a
	S	87	4.7a	7.3a	35.0	12.5	4.8	2.1	109.2b
6	R	39	4.2b	8.9b	28.7	16.8	5.7	1.7	106.8b
	S	52	5.1a	10.4a	32.2	15.5	5.9	1.7	107.4a

#### *Fhb1* plus *QFhs.nau-2DL*

For populations 3, 4, and 6, the double QTL combination RR (*Fhb1, QFhs.nau-2DL* respectively) was always lowest or in the lowest significant (*P* ≤ 0.05) grouping in both years for DON (Table 5). Conversely the double susceptible (SS) lines always had the highest mean DON, or were in the statistical grouping with the highest DON for both years (Table 5). In 2012 in all populations, RR and SS were significantly (*P* ≤ 0.05) different (Table 5). In 2011 populations 4 and 6 RR and SS lines mean DON were also significantly (*P* ≤ 0.05) different. For population 3, lines resistant at *Fhb1* only (RS lines) were significantly (*P* ≤ 0.05) different from SS lines (Table 5). No allele combination had any significant effect on DON in population 2 in either year (Table 5). For populations 3, 4, and 6 the reduction in DON in RR vs. SS lines was 10.2, 26.8, and 33.9% respectively in 2011 (Table 5). The same trend was seen in 2012 with 29.3, 43.9, and 46.7% reductions respectively (Table 5). It seemed that the resistances were also additive in 2012, and the greater individual effects of *Fhb1* and especially *QFhs.nau-2DL* in the milder epidemic in that year combined to produce a greater effect in RR lines compared to 2011. The remarkable consistency in effect on DON was also seen for FDK. In populations 3, 4, and 6 in 2011 the reductions were 19.3, 30.5, and 33.9% respectively (Table 5). In 2012 they were 10.7, 29.6, and 30.4% respectively (Table 5). In neither year was a significant (*P* < 0.05) difference in FDK seen between any of the allele combinations for population 2 (Table 5). For incidence, severity and index, more significant differences were seen among the four allele combinations than were seen when looking at the QTL individually (Tables 3–5). Significant (*P* < 0.05) differences in incidence were seen in both 2011 and 2012 in populations 3 and 4. In 2011 significant (*P* < 0.05) differences in severity were seen among populations 2, 3, and 6 (Table 5), while in 2012 significant (*P* < 0.05) differences were again seen in populations 2 and 3. In population 3 in 2012 the lowest severity seen in SR lines, significantly (*P* < 0.05) different only from SS lines. The detailed pattern for index was similarly complex, varying by population and year (Table 5). Ratings were more consistent between the years in populations 4 and 6. For both populations, RS was usually next lowest to RR and often not significantly (*P* < 0.05) different. Ratings over years were less consistent in Populations 2 and 6 (Table 5).

**Table 5:** **Means for FHB traits evaluated according to the presence of resistance (R) or susceptible (S) alleles at two QTL (*Fhb1* and *QFhs.nau-2DL* respectively), for four F_2_ derived wheat populations, Lexington, KY, 2011 and 2012**.

**Population**	**Allele**	**N**	**DON**	**FDK**	**Inc**	**Sev**	**Ind**	**Rating**	**Heading**
			**ppm**	**___________%___________**				**0–9**	**Julian days**
**2011**									
2	RR	18	13.4	19.8	58.3	48.6b	28.9ab	3.6b	134.8a
	RS	22	13.8	24.5	56.5	53.7a	30.9a	4.7a	133.0b
	SR	17	13.8	21.4	52.3	45.1b	24.6b	3.6b	134.7a
	SS	21	14.8	23.8	52.9	49.0b	26.6ab	3.8b	134.6a
3	RR	19	12.3ab	19.7b	61.1a	45.5b	28.1	4.2	134.7
	RS	20	11.8b	26.2a	57.8ab	50.9a	30.1	4.4	133.5
	SR	15	13.2ab	22.0ab	50.8b	51.6a	26.5	4.1	133.3
	SS	25	13.7a	24.4a	60.4a	51.4a	31.5	4.2	134.4
4	RR	24	12.0b	20.7c	45.9b	46.4	21.9b	3.6c	133.6
	RS	43	12.8b	20.6b	50.7b	46.6	24.2ab	3.8bc	134.1
	SR	20	15.4a	22.7b	57.1a	47.5	27.7a	4.3ab	134.6
	SS	44	16.4a	29.8a	50.3b	48.1	24.7ab	4.2a	134.4
6	RR	16	10.9c	18.7c	40.6	34.3b	14.8b	2.9c	131.5
	RS	29	11.0c	23.7b	43.6	36.8b	16.8ab	3.7b	131.9
	SR	23	14.0b	23.1bc	45.6	34.8b	16.4ab	4.4a	131.0
	SS	23	16.1a	28.3a	45.0	41.2a	19.0a	4.4a	131.5
**2012**									
2	RR	18	8.7	9.8	52.9	20.3a	12.5a	3.9a	112.6a
	RS	22	7.8	9.3	50.5	16.8b	8.5b	2.9b	108.3b
	SR	17	8.4	9.7	53.1	19.5ab	11.4a	3.7a	113.0a
	SS	21	8.3	9.9	54.3	20.9a	12.6a	3.7a	113.2a
3	RR	19	2.9b	7.5b	30.1b	13.7ab	5.2ab	2.0	110.4a
	RS	20	3.5ab	9.5a	34.6ab	12.4b	4.5b	2.0	109.4b
	SR	15	3.2b	7.7b	28.8b	12.3b	3.8b	2.1	110.2a
	SS	25	4.1a	8.4b	40.2a	14.8a	6.3a	2.3	109.6b
4	RR	24	3.2c	5.7c	27.9b	12.9	4.4	1.9b	109.7a
	RS	43	3.7bc	6.5b	33.5a	12.3	4.5	1.8b	109.0b
	SR	20	4.6b	6.7b	38.0a	13.2	5.7	2.6a	109.4a
	SS	44	5.7a	8.1a	36.4a	12.6	5.1	2.4a	109.5a
6	RR	16	3.2c	7.8c	28.1	16.5	6.0	1.3c	106.7b
	RS	29	4.3b	9.7b	32.9	15.2	5.9	1.5bc	107.5a
	SR	23	4.8b	9.6b	29.1	17.0	5.5	2.0a	106.9b
	SS	23	6.0a	11.2a	31.2	15.9	5.9	1.9ab	107.2ab

#### Resistance alleles in highly resistant lines

We focused on the 20 most resistant lines in each population in each year with rankings based on DON concentration in 2011 and 2012. We especially looked at those lines that were the lowest 20 in both years

Population 2, in which the average impact of either QTL was not significant (*P* < 0.05), presented a neutral picture consistent with the lack of impact of either QTL. Of the 20 lowest DON lines in each year, 12 were common to both years; 3 of these had RR alleles, 3 had RS, 3 had SR and 3 had the SS genotype (Table 6). Line 274508 from this population, with RS alleles, had the lowest mean DON for both years, 4.5 ppm. Populations 3 and 4 had 5 and 7 RR lines common to both years respectively, the highest numbers of RR lines common to both years (Table 6). The high number of RR lines was interesting given the lack of significant (*P* < 0.05) differences between RR and RS lines in both populations in both years (Table 5). Population 6 had just 2 RR lines consistently in the lowest 20 for DON but 6 RS lines, no SR lines and 1 SS line (Table 6). The predominance of RS genotypes among consistently highly ranked lines was unexpected. Population 6 was the only population that in 2012 had showed a significant (*P* < 0.05) improvement of RR over RS lines (Table 5). Surprisingly for a population that showed a significant (*P* < 0.05) average benefit from both QTL, but not for a population with so mostly RS genotypes in the lowest 20, the lowest line for mean DON both years was an RS line.

**Table 6:** **Number of the 20 lowest DON F_2_ - derived lines from four wheat populations with resistance alleles at either, both, or neither of two QTL: *Fhb1* and *2DL***.

**Year**	**QTL**	**Population**			
		**2**	**3**	**4**	**6**
**2011**					
	*Fhb1* only	5	5	7	12
	*2DL* only	3	3	2	1
	Both	5	7	9	5
	Neither	7	5	2	2
**2012**					
	*Fhb1* only	5	5	5	8
	*2DL* only	6	2	2	4
	Both	4	7	9	6
	Neither	5	6	4	2
**COMMON TO BOTH YEARS**					
	*Fhb1* only	3	3	3	6
	*2DL* only	3	0	2	0
	Both	3	5	7	2
	Neither	3	2	0	1

Lines were evaluated in inoculated, irrigated scab nurseries 2011 and 2012, Lexington, KY.

#### QTL x environment interaction in highly resistant lines

In populations 2, 3, and 4, where no QTL x environment interactions were seen, the majority of the two-resistance-allele lines that were among the lowest 20 DON lines in each year were common to both years (Table 6). In population 6, where QTL x environment interaction was seen, 5 and 6 RR lines were among the lowest 20 in 2011 and 2012 respectively, and of these, 2 were common to both years (Table 6). The consistency of low DON SS lines also varied by population. For example, in 2011 and 2012, 7 and 5 of the lowest DON lines in population 2 were SS and of these 3 were common to both years. On the other hand, in population 3, 5, and 6 of the top 20 lines in 2011 and 2012 respectively had neither resistance allele and 2 of these were common to both years.

#### Low DON segregants

Segregants with low DON were observed in all populations, but were much more frequent in Population 3 where 19 lines were found to have DON levels lower than the resistant parent VA01W-476, in the 2012 scab nursery. Across all populations there were 31 lines with numerically lower DON than VA01W-476: 4, 4, 19, and 4 in populations 2, 3, 4, and 6 respectively (data not shown). Seventeen lines had DON levels that were more than two standard errors lower than the 1.6 ppm measured in VA01W-476.

#### NIR predictions

Correlations between FDKNIR and actual FDK in this study were 0.49 for all populations combined (Table 7) with highest value in Population 6 (*r* = 0.73; data not shown). Correlations between DONNIR and FDK were 0.53 (Table 7). Correlations between DONNIR and DON ranged from 0.55 for population 2 to 0.82 for population 6 (data not shown), with a value of 0.63 among all populations combined (Table 7). Interestingly, FDKNIR was better correlated to DON (0.64) than FDK itself (0.47; Table 7). Visual ratings also proved to be predictive of DON overall, with a correlation of 0.67 (Table 7). The correlations indicate NIR of grain and even visual ratings would have been good predictors of FDK and DON values of the soft red winter wheat populations in this study. We wanted to see how many of the lowest DON lines identified by GC-MS would also have been selected using NIR or ratings. For this comparison of 2 year means, in every population DONNIR identified the highest, or joint highest, number of the 20 lowest DON lines compared to FDK, FDKNIR, rating, and incidence, severity or index (Table 8). The usefulness of the DON-substitute measurements varied between populations (Table 8).

**Table 7:** **Pearson correlation coefficients among height, heading date (Heading) and disease traits [Rating, Incidence, Severity, FHB index, *Fusarium* damaged kernels (FDK), deoxynivalenol (DON), and FDK and DON estimated by NIR]**.

	**FDK**	**DONNIR**	**FDKNIR**	**Incidence**	**Severity**	**Index**	**Rating**	**Heading**	**Height**
DON	0.47 < 0.0001	0.63 < 0.0001	0.64 < 0.0001	0.45 < 0.0001	0.54 < 0.0001	0.51 < 0.0001	0.67 < 0.0001	0.38 < 0.0001	−0.048 ns
FDK		0.53 < 0.0001	0.48 < 0.0001	0.25 < 0.0001	0.33 < 0.0001	0.31 < 0.0001	0.43 < 0.0001	0.22 < 0.0001	−0.40 < 0.0001
DONNIR			0.84 < 0.0001	0.29 < 0.0001	0.44 < 0.0001	0.37 < 0.0001	0.52 < 0.0001	0.12 < 0.05	−0.22 < 0.0001
FDKNIR				0.32 < 0.0001	0.45 < 0.0001	0.38 < 0.0001	0.50 < 0.0001	0.32 < 0.0001	−0.24 < 0.0001
Incidence					0.66 < 0.0001	0.88 < 0.0001	0.65 < 0.0001	0.48 < 0.0001	−0.10 < 0.05
Severity						0.87 < 0.0001	0.71 < 0.0001	0.48 < 0.0001	−0.064 ns
Index							0.71 < 0.0001	0.53 < 0.0001	−0.088 ns
Rating								0.31 < 0.0001	−0.12 < 0.05
Heading									−0.073 ns

Data comprised 2 year entry means in four F_2_-derived wheat populations, Lexington, KY 2011–2012.

**Table 8:** Number of the 20 lowest DON F_2_—derived lines from four wheat populations identified using FDK, DONNIR, FDKNIR, scab rating, incidence, severity, and index^*^.

**Population**	**FDK**	**DON**	**FDK**	**Rating**	**Incidence**	**Severity**	**Index**
		**NIR**	**NIR**				
2	11	12	9	10	3	12	7
3	13	14	12	7	7	10	7
4	9	11	10	11	8	11	9
6	6	10	10	9	5	8	8

* FDK, Fusarium damaged kernels; DONNIR, DON predicted by NIR; FDKNIR, FDK predicted by NIR.

#### Agronomic performance of F_2_ and BC_1_F_1_-derived lines

Yield trial data from BC_1_F_1_ derived line tests at Lexington and Princeton in 2011 and 2012 is presented in Table 9. Yield trial data for BC_1_F_1_ and F_2_ derived lines in 2012 is shown in Tables 10, 11. In population 2 in 2012, 23% of the 86 lines tested were not significantly different from the high yielding commercial cultivars that were used as checks (data not shown) a number similar to that seen for BC_1_F_1_ derived lines in both years (Table 9). The highest yielding lines were all BC_1_F_1_ derived (Table 10), while F_2_ derived lines did not fare as well. The backcross lines were also screened in the scab nursery in 2012. Several of the lines at the very top of the yield trial also performed well in the scab nursery, with DON concentrations of 8 ppm for example, in comparison to the susceptible recurrent parent in which the DON level was 15.9 ppm (Table 11). These results indicate that it may be possible to combine scab resistance and superior agronomic performance even in populations in which the apparent effect of the QTL is muted or negligible.

**Table 9:** **Two year mean yield and test weight of BC_1_F_1_ derived wheat lines, total number of lines and percentage of lines with yields not significantly different from checks, Lexington and Princeton, KY, 2011–2012**.

**Population**	**Statistic**	**Yield kg ha^−1^**	**Test Weight kg hL^−1^**	**Number of lines**	**Lines with yield n.s. different from checks %**
2	Mean	4241	73.3	65	23.3
	Range	3464–4567	68.6–76.3		
	CV	8.7	2.3		
3	Mean	3426	70.3	23	21.9
	Range	2797–3803	64.7–71.9		
	CV	15.1	19.5		
4	Mean	3956	77.5	41	13.3
	Range	3347–4615	75.6–79.0		
	CV	10.6	2.3		
6	Mean	4174	74.2	32	12.5
	Range	3600–4792	69.6–77.0		
	CV	13.9	9.9		

**Table 10:** **Mean yield and test weight of BC_1_F_1_ and F_2_ derived wheat lines, total number of lines and percentage of lines with yields not significantly different from checks, Lexington and Princeton, KY, 2012**.

**Population**	**Statistic**	**BC_1_F_1_Yield kg ha^−1^**	**F_2_Yield kg ha^−1^**	**BC_1_F_1_Test Weight kg hL^−1^**	**F_2_ Test Weight kg hL^−1^**	**Number of BC_1_F_1_ lines**	**Number of F_2_ lines**	**BC_1_F_1_Lines with yield n.s. different from checks %**	**F_2_Lines with yield n.s. different from checks %**
2	Mean	4668	4291	75.6	77.0	65	21	76.7	57.1
	Lowest	4179	3553	72.7	75.1				
	Highest	5261	4950	78.2	78.5				
	CV	6.6	11.1	1.6	1.5				
3	Mean	3940	3255	74.5	76.3	23	5	62.5	60.0
	Lowest	3073	3145	70.3	75.7				
	Highest	4401	3462	77.0	77.1				
	CV	8.8	11.9	1.5	0.5				
4	Mean	3956	4014	77.5	77.2	41	44	50.0	40.9
	Lowest	3347	3529	75.6	74.6				
	Highest	4615	5213	79.0	78.9				
	CV	8.0	10.6	1.3	1.3				
6	Mean	4407	4237	76.2	76.9	32	36	73.6	72.2
	Lowest	3837	3318	73.2	74.7				
	Highest	4997	4926	78.4	78.4				
	CV	7.3	8.0	1.5	1.2				

**Table 11:** **Yield, test weight and DON concentrations of top yielding backcross-derived and F2 derived lines in population 2 along with several commercial checks and donor and recurrent parents**.

**Entry**	**Yield (kg ha^−1^)**	**TESTWT (kg hL^−1^)**	**DON (ppm)**
BC_1_–803	5261	75.3	8.1
BC_1_–023	5252	73.6	20.5
BC_1_–056	5188	75.3	13.2
BC_1_–036	5183	76.8	7.6
BC_1_–048	5133	74.0	7.3
BC_1_–070	5125	73.3	9.7
BC_1_–028	5058	74.0	7.6
BC_1_–520	5028	75.5	10.1
BC_1_–054	5023	73.5	5.8
Pembroke	5016	75.2	n.d.
BC_1_–074	4974	74.1	10.7
BC_1_–035	4969	72.7	10.1
SS 8302	4958	74.7	n.d.
F_2_-216	4950	78.5	n.d.
Yield statistics: CV 9.5% LSD(0.05) 901 kg ha^−1^			
KY97-0321-05-2	Recurrent parent		15.9
VA01W-476	Donor parent		1.6

n.d., Not determined.

Two year yield averages for Populations 2, 3, 4, and 6 were close to 4237 kg ha^−1^ (Sarti-Dvorjak, 2014). This value is numerically but not significantly lower than the yield average of the check cultivars (4371 kg ha^−1^). When measured in yield plots, F_2_ derived lines in Populations 2, 4, and 6 were 10, 9, and 13% taller than their respective susceptible parent's average height (Sarti-Dvorjak, 2014). Both F_2_ and BC_1_F_1_ populations were effective sources of useful segregates, though it varied by population. In population 2, only one F_2_-derived line was competitive with the yields of commercial check cultivars while maintaining an acceptably low DON concentration, in contrast to 11 BC_1_F_1_-derived lines that met these criteria (Table 11). However, in population 6 the top yielding lines in the same LSD group as the checks were BC_1_F_1_ and F_2_ derived in roughly equal numbers (9 and 11 respectively, data not shown). In population 3 all of the top yielding experimental lines were BC—derived, while in population 4, there were 7 F_2_ derived and 4 BC_1_F_1_—derived lines that out yielded the commercial checks.

#### Milling and baking quality

In a previous study of five SRW populations, Balut et al. (2013) found little impact of resistance alleles at either *Fhb1* or *QFhs.nau-2DL* on milling and baking quality traits. In this study, in all populations, the BC_1_F_1_ derived lines have better quality scored than the F_2_ derived lines as one would expect with 75 vs. 50% of recurrent parent in the pedigree (Table 12). The challenge in this instance is finding lines that combine superior agronomic performance with acceptable FHB resistance and acceptable milling and baking quality. In population 2, there were eight such lines selected for comparison because they had an average FDK of 10.0 vs. 9.6% for the highly resistant donor parent. The yield and test weight averages of these lines were equivalent to those of the checks (4375 vs. 4419 kg ha^−1^, and 73.5 vs. 72.8 kg hl^−1^) and the quality, based on the traits listed in Table 11, was actually slightly higher than that of the check cultivars (data not shown).

**Table 12:** **Mean values of milling and baking quality traits from wet lab analyses and predicted by NIR from BC_1_F_1_ and F_2_—derived wheat populations, parents, and commercial check cultivars grown at Lexington, KY 2012**.

**Population**	**Units**	**2**		**3**		**4**		**6**		**Parents**		**Commercial Mean**
		**BC_1_**	**F_2_**	**BC_1_**	**F_2_**	**BC_1_**	**F_2_**	**BC_1_**	**F_2_**	**Donor**	**Recurrent**	
Whole grain protein	%	10.4	11.0	10.9	12.1	11.8	12.2	11.5	12.0	13.2	10.2	
Whole grain hardness	0–100	33.9	32.7	26.5	33.0	35.3	33.4	32.4	32.7	32.0	28.1	
Flour yield	%	68.1	66.8	68.3	65.4	65.6	63.9	66.3	65.8	62.9	68.5	68.3
Flour softness equivalent	%	54.0	51.8	51.9	50.3	51.8	47.6	51.7	49.3	47.0	57.0	56.5
Flour protein	%	8.7	9.2	8.8	10.0	10.0	10.3	10.0	10.2	10.8	8.5	8.4
Lactic acid SRC	%	91.7	95.2	87.0	93.0	103.4	86.9	103.4	101.0	100.1	94.6	93.2
Sucrose SRC	%	88.4	89.1	86.1	90.0	91.3	91.0	90.2	89.3	88.8	89.5	88.6
Water SRC	%	57.7	58.2	55.1	57.2	58.0	58.3	56.9	57.5	59.4	56.3	55.6
Sodium carbonate SRC	%	68.1	68.5	66.5	68.7	68.9	69.2	66.3	66.5	68.9	66.7	66.4
Cookie diameter	cm	18.6	18.3	18.5	18.0	18.0	17.8	18.0	17.9	17.7	18.7	18.7

## Discussion

The high DON h^2^ estimates seen in these populations were similar to results reported by Balut et al. (2013) but lower than Agostinelli et al. (2012). FDK h^2^ were lower than estimates reported by Agostinelli et al. (2012) and Balut et al. (2013), which exceeded 0.60.

Resistance alleles and the interaction among FHB resistance QTL have distinct behavior in different genetic backgrounds in wheat. The best-validated gene for FHB resistance, *Fhb1* on chromosome 3BS, showed an average reduction of 17.5% in DON, effecting significant improvement of FHB resistance in 3 of 4 populations. This is the first study to validate *QFhs.nau-2DL* resistance using homozygous lines. In this study the effect was modest, significant in 2 of 4 populations and reducing DON 6.7% overall. *QFhs.nau-2DL* seemed to combine well with *Fhb1* however. The combined resistances reduced DON 25.5% across all populations.

Previous investigators have questioned whether exotic QTL will provide sufficient resistance to progeny in the absence of native resistance (Balut et al., 2013). The recurrent parent in Population 2, KY97C-0321-05-2, had been increased for possible release for its yield potential, but FHB susceptibility derailed this effort. Thus, it seemed the perfect candidate to ascertain whether backcrossed resistance QTL would lead to adequate levels of FHB resistance. While the *overall* impact of either QTL was negligible in this genetic background (Tables 3–5) breeders are always looking for rare segregants that perform the best in each population. In assessing each of the four populations it made sense to look beyond the average effect, at individual F_2_ lines. At the outset of this study, it was our assumption that it would be difficult, if not impossible, to identify FHB-resistant segregants from populations in which the expression of the introgressed QTL was muted. In contrast to our expectations, resistant segregants were seen for DON accumulation, even in population 2 as well as the other populations. This underscores our assertion that usable segregants can be recovered in many recalcitrant populations. Furthermore, we found that there were individual lines in all populations in which agronomic fitness was combined with acceptable levels of FHB resistance. In population 2, for example, such segregants were identified with and without the R alleles at one or both QTL, even though the only apparent native resistance came from the donor parent. In sum, our results give cause for optimism concerning the utility of even the most intractable populations in FHB resistance breeding.

The key message that emerges from this complex picture is that in every population there were lines with low DON levels both years, under very heavy (2011) and rather light (2012) scab pressure. These results indicate that if a population with a scab resistant and scab resistance QTL-containing parent has the potential to deliver agronomically promising breeding lines it is critical to evaluate all of the progeny from crosses that may appear to be generally intransigent for scab resistance.

Previous studies of *Fhb1* and *QFhs.nau-2DL* in our lab have revealed significant QTL by year interaction in several populations (Agostinelli et al., 2012; Balut et al., 2013). The nature of such interaction is important: significant changes in rank (crossovers) across environments would have a major impact on marker-assisted selection and reduce the utility of the QTL derived resistance. However, significant QTL x environment interaction does not preclude the existence of lines with consistent QTL effects in very different environments. As noted earlier, the donor parent, VA01W-476, probably contributed native resistance as well as *Fhb1* and *QFhs.nau-2DL* and it is possible this was the source of resistance that consistently lowered DON in low DON SS lines.

Scab resistant progeny were found only through arduous phenotypic screening and further investigation and optimization of methods of efficiently collecting phenotypic scab data are needed. Our results also assert the importance of grain-based measures of FHB resistance. It has long been our assumption that the time and labor-consuming activities of assessing incidence and severity are of limited value if they are not effective in predicting FDK and DON, because FDK and DON are the best indicators of loss to growers, processors and end users. In this study, overall the best predictor of DON was, surprisingly, FHB rating (0.67; Table 7). This 0–9 “eyeball” measure is taken quickly and integrates incidence and severity, though it is essential that the ratings are completed by only one individual for a given test. A close second was FDKNIR (0.64; Table 7), which is a compelling reason to continue to look at NIR as a way of estimating scab damage. Initially, the purpose of this comparison was to estimate the correlation between scab damage and DON levels with NIR predictions, to determine whether NIR might eventually replace expensive, time consuming and destructive techniques like DON gas chromatography mass spectrometry (GC-MS). Alternatively, NIR might be useful to prescreen samples before using GC-MS. Previous studies in our lab have shown strong positive correlations between FDK and DON values measured with traditional methods and NIR estimations. Balut et al. (2013) reported FDK—FDKNIR correlations of 0.70 and 0.73 and DON—DONNIR of 0.56 and 0.63 in 2010 and 2011, respectively. Tibola et al. (2010) tested the ability of NIR to predict DON levels in both 125 grams whole grain and milled samples in the Southern Brazil and reported 0.89 and 0.91 coefficient of determination (*R*^2^). While DONNIR was not entirely successful as a replacement for GC-MS DON measurement in the study described here, it did perform better than FDK in every population. In breeding populations that would be screened by FDK but not GC-MS, substitution of DONNIR should continue to be investigated.

Finally, we were interested in the value of BC_1_F_1_ vs. F_2_—derived populations in generating usable progeny. In this study both kinds of populations were effective in this regard, though it was clear when we looked at milling and baking quality traits that BC_1_F_1_—derived populations had the edge. This fact is a good reason for breeders to consider creating BC_1_F_1_ populations for routine breeding and extraction of inbred lines in addition to pursuing the more customary route through selection in F_2_ populations.

## Author contributions

DV overall responsibility for design of the study and resultant manuscript, contributed text, data analysis, table preparation, editing, and revising. AC primary responsibility for writing text and preparing tables, data analysis, revising, editing. DS carried out the research for her PhD; contributed data and text, revised as needed. GB: carried out genotyping; contributed text describing same, revising as needed. BB: conducted milling baking analyses; contributed text describing same, revising as needed. YD conducted mycotoxin analyses; contributed text describing same, revising as needed. All authors participated in discussions that related to the design of the study and all contributed significant intellectual content to the manuscript.

### Conflict of interest statement

The authors declare that the research was conducted in the absence of any commercial or financial relationships that could be construed as a potential conflict of interest.
